# Understanding ADC variation by fat content effect using a dual-function MRI phantom

**DOI:** 10.1186/s41747-023-00414-0

**Published:** 2024-02-13

**Authors:** Yi-Jui Liu, Tung-Sheng Tsai, Ya-Hui Li, Jo-Hua Peng, Hing-Chiu Chang, Hsu-Hsia Peng, Ying-Chieh Lee, Tung-Yang Lee, Chang-Hsien Liou, Tz-Feng Lin, Fatt-Yang Chew, Ruey-Hwang Chou, Chun-Jung Juan

**Affiliations:** 1 Department of Automatic Control Engineering, Feng Chia University, Taichung, Taiwan, Republic of China; 2 Ph.D. Program in Electrical and Communication Engineering, Feng Chia University, Taichung, Taiwan, Republic of China; 3 Department of Medical Imaging, China Medical University Hsinchu Hospital, 199, Sec. 1, Xinglong Rd., Zhubei City, Hsinchu County 302 Taiwan, Republic of China; 4 Department of Biomedical Engineering and Environmental Sciences, National Tsing Hua University, Hsinchu, Taiwan, Republic of China; 5 Department of Biomedical Engineering, Chinese University of Hong Kong, Hong Kong, China; 6 Master’s Program of Biomedical Informatics and Biomedical Engineering, Feng Chia University, Taichung, Taiwan, Republic of China; 7 Cheng Ching Hospital, Taichung, Taiwan, Republic of China; 8 Institute of Nuclear Engineering and Science, National Tsing Hua University, Hsinchu, Taiwan, Republic of China; 9 Department of Fiber and Composite Materials, Feng Chia University, Taichung, Taiwan, Republic of China; 10 Department of Medical Imaging, China Medical University Hospital, Taichung, Taiwan, Republic of China; 11 Department of Radiology, School of Medicine, China Medical University, Taichung, Taiwan, Republic of China; 12 Graduate Institute of Biomedical Sciences, China Medical University, Taichung, 406 Taiwan, Republic of China; 13 Center for Molecular Medicine, China Medical University Hospital, Taichung, Taiwan, Republic of China; 14 Department of Medical Laboratory and Biotechnology, Asia University, Taichung, Taiwan, Republic of China

**Keywords:** Adipose tissue, Diffusion magnetic resonance imaging, Phantoms (imaging), Quality assurance (healthcare), Quality control

## Abstract

**Background:**

A dual-function phantom designed to quantify the apparent diffusion coefficient (ADC) in different fat contents (FCs) and glass bead densities (GBDs) to simulate the human tissues has not been documented yet. We propose a dual-function phantom to quantify the FC and to measure the ADC at different FCs and different GBDs.

**Methods:**

A fat-containing diffusion phantom comprised by 30 glass-bead-containing fat-water emulsions consisting of six different FCs (0, 10, 20, 30, 40, and 50%) multiplied by five different GBDs (0, 0.1, 0.25, 0.5, and 1.0 g/50 mL). The FC and ADC were measured by the “iterative decomposition of water and fat with echo asymmetry and least squares estimation-IQ,” IDEAL-IQ, and single-shot echo-planar diffusion-weighted imaging, SS-EP-DWI, sequences, respectively. Linear regression analysis was used to evaluate the relationship among the fat fraction (FF) measured by IDEAL-IQ, GBD, and ADC.

**Results:**

The ADC was significantly, negatively, and linearly associated with the FF (the linear slope ranged from -0.005 to -0.017, R^2^ = 0.925 to 0.986, all *p* < 0.001). The slope of the linear relationship between the ADC and the FF, however, varied among different GBDs (the higher the GBD, the lower the slope). ADCs among emulsions across different GBDs and FFs were overlapped. Emulsions with low GBDs plus high FFs shared a same lower ADC range with those with median or high GBDs plus median or lower FFs.

**Conclusions:**

A novel dual-function phantom simulating the human tissues allowed to quantify the influence of FC and GBD on ADC.

**Relevance statement:**

The study developed an innovative dual-function MRI phantom to explore the impact of FC on ADC variation that can affect clinical results. The results revealed the superimposed effect on FF and GBD density on ADC measurements.

**Key points:**

• A dual-function phantom made of glass bead density (GBD) and fat fraction (FF) emulsion has been developed.

• Apparent diffusion coefficient (ADC) values are determined by GBD and FF.

• The dual-function phantom showed the mutual ADC addition between FF and GBD.

**Graphical Abstract:**

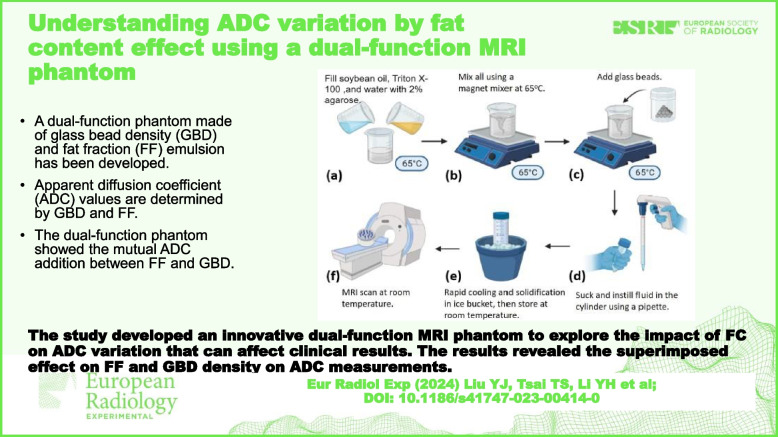

## Background

Magnetic resonance imaging (MRI) has been recognized to provide quantitative imaging biomarkers [1] by measuring tissue characteristics and function, including but not limited to brain volume [2], tumor volume [3], functional network connectivity [4], fat content (FC) [5], and diffusivity of tissue water [6]. Diffusion-weighted imaging (DWI) allows measuring proton diffusivity by calculating the apparent diffusion coefficient (ADC) [6], while the “iterative decomposition of water and fat with echo asymmetry and least squares estimation-IQ” (IDEAL-IQ) sequence allows measuring the FC of tissue [7].

In order to increase the reliability and comparability of data gathered from MRI biomarkers, standardization of imaging protocols and calibration of measures using phantoms are necessary to validate the accuracy of these measures *in vivo* [8]. Therefore, the Radiological Society of North America established the Quantitative Imaging Biomarkers Alliance and quantitative imaging protocols, phantoms, and technical standards documents [9]. There are many kinds of phantoms that provide different functions. For example, the traditionally used American College of Radiology phantom is a system phantom allowing evaluation of the image quality and comparison across geometric distortion, slice positioning, thickness accuracy, high-contrast spatial resolution, intensity uniformity, ghosting artifact, and low-contrast object detectability [10]. Other phantoms include but not limited to diffusion phantom, which is designed for quantifying the ADC measurement [11], and water-fat phantom, which is used for monitoring and calibrating fat fraction measurement [12, 13].

Recently, various ADC phantoms made from tissue-equivalent materials have been developed to simulate the different forms of restricted Brownian motion of water. These phantoms serve multiple purposes, including optimizing DWI protocols and evaluating the accuracy and reproducibility of ADC measurements. Materials used in ADC phantoms can include varying concentrations of high-viscosity gelatinous substances to simulate different degrees of restricted Brownian motion of water, such as polyvinylpyrrolidone [14], polyethylene glycol [15, 16], polyacrylamide [17, 18], sucrose [19–21], and agarose [17, 19]. Another approach involves using various densities of microparticles to mimic cell-occupied spaces and increase barrier concentration, resulting in different levels of restricted Brownian motion of water [11, 22].

However, a single-function phantom is not sufficient to fit real-world situations. For example, DWI might be performed in fat-containing tissue such as breast [23], salivary gland [24], and vertebra and measurement of the ADC value might be influenced by the content of fat in the breast. Taking breast lesion for example, a high ADC is interpreted as a sign of benign nature of the lesion, while a low ADC is classified as a sign of malignancy [25]. Subsequently, a fatty mass, such as fat necrosis, might be mistakenly interpreted as a malignancy due to its low ADC if the FC is not taken into account [25]. To avoid mistakenly interpreting a fat-containing mass with low ADC as a malignant lesion, simultaneous measures of both ADC and FC is crucial rather than single measure of ADC.

To the best of knowledge, a dual-function phantom designed to quantify the ADC in different FCs and different glass bead densities (GBDs) to simulate the human tissue has not been documented yet. In this study, we designed a novel dual-function phantom to allow us to quantify the FC and to measure the ADC at different FCs and different GBDs.

## Methods

### Phantom design

The composition of each cylindrical cone of a fat-containing diffusion phantom, which consisted of six different FCs (0, 10, 20, 30, 40, and 50%) *versus* five different GBDs (0, 0.1, 0.25, 0.5, and 1.0 g/50 mL), used in this study is shown in Table 1. In this phantom, various percentages of water and soybean oil emulsified by an emulsifying agent (Triton X-100) [26] were mixed with various GBDs (#K37, 3M microspheres, Easy Composites Ltd., Stoke, UK) [11]. Agarose was added into the emulsion to serve as a coagulating agent to prevent dissociation of the water-oil emulsion on the one hand and sinking/suspension of glass particles on the other hand [11].

**Table 1:** Composition of the proposed fat-containing diffusion phantom

FC (%)	Soybean oil (V%)	Triton X-100 (V%)	Water (V%)	GBD (g/50 mL)				
0	0.0	0.0	100					
10	10.0	0.4	89.6					
20	19.8	0.8	79.4	0.00	0.10	0.25	0.50	1.00
30	29.6	1.2	69.2					
40	39.4	1.6	59.0					
50	49.0	2.0	49.0					

*FC* Fat content (%), *GBD* Glass bead density (g/50 mL), *V%* Volume percentage

**Fig. 1:**
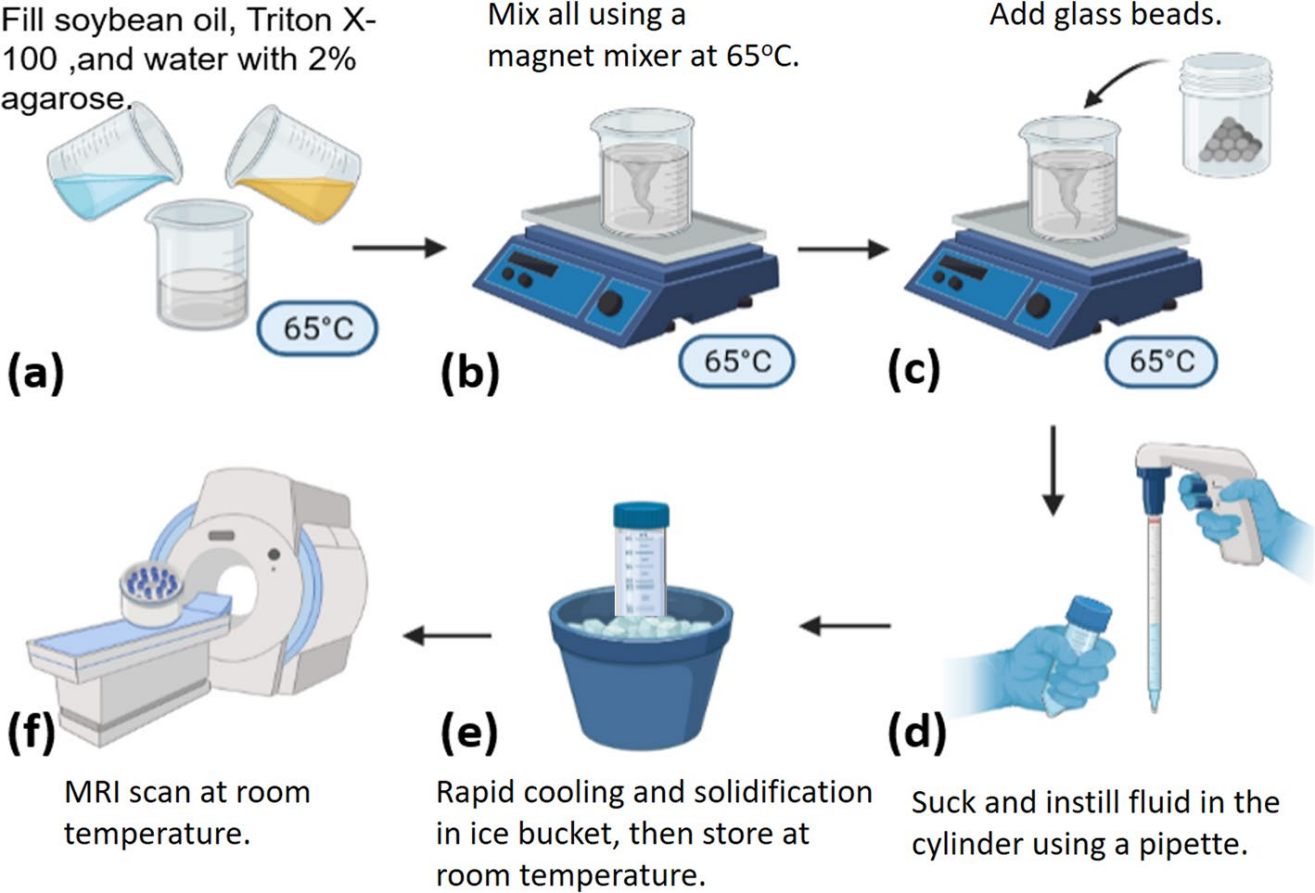
The pipeline illustrating the processes for producing a fat-containing diffusion phantom. Soybean oil (yellow) and water (blue) were mixed together with a nonionic surfactant (Triton X-100) and a coagulant (2% agarose) at 65 °C (**a**). Fluid was stirred at a speed of 700 revolution per min (RPM) for 2 min and then at a speed of 1,150 RPM for 5 min at 65 °C (**b**). Glass beads were added with the fluid continuously stirred at a speed of 1,150 RPM for 2 min at 65 °C (**c**). The mixture was sucked out and gradually instilled into a cylindrical cone (50 mL) using a pipette (**d**). The cylindrical cone was put in the ice bucket to cool down rapidly to turn the sticky fluid into jelly to keep the homogeneity of all components (**e**). A phantom comprised by 30 glass-bead-containing fat-water emulsions consisting of six fat fractions multiplied by five glass bead densities was produced and put into a plastic container (**f**)

The pipeline demonstrating the processes for preparing the proposed phantom is shown in Fig. 1. First of all, soybean oil and water at a certain ratio were mixed together with a nonionic surfactant (Triton X-100, Sigma-Aldrich, Massachusetts, USA) and a coagulant (2% agarose) at 65 °C (Fig. 1a). Second, fluid was stirred at a speed of 700 revolution per minute (RPM) for 2 min and then at a speed of 1,150 RPM for 5 min at 65 °C (Fig. 1b). Third, glass beads at a certain density were added with the fluid continuously stirred at a speed of 1,150 RPM for 2 min at 65 °C (Fig. 1c). Fourth, the mixture was sucked out and gradually instilled into a cylindrical cone (50 mL) using a pipette (Fig. 1d). Fifth, the cylindrical cone was put in the ice bucket to cool down rapidly. The sticky fluid turned to jelly immediately to keep the homogeneity of all components (Fig. 1e). Sixth, a phantom comprised by 30 glass-bead-containing fat-water emulsions consisting of six FFs multiplied by five GBDs was produced and put into a plastic container for MRI scans (Fig. 1f). Finally, a total of three repetitions, each containing 30 cylindrical cones, were prepared, respectively. The phantoms were stored in the MRI scan room for 1 day to allow their temperature to equilibrate with that of the MRI room. The MRI scan room temperature was maintained at a stable 20°C by the air conditioning system, with temperature monitoring conducted via the operation console of MRI vendor.

### MRI protocols

All images were acquired using a 1.5-T scanner (Signa MR450, General Electric Healthcare, Chicago, USA). IDEAL-IQ sequence was applied to quantify the FC of the phantom [8]. The IDEAL method [27] was a three-dimensional fast spoiled gradient-echo sequence employing a six-echo acquisition (1.1 to 6.38 ms) with imaging parameters including repetition time of 19.6 ms, field of view of 220 × 220 mm, matrix size of 128 × 128, bandwidth of 90.91 kHz, flip angle of 5°, number of excitations 4, and slice thickness of 10 mm. DWI was performed using a single-shot echo-planar imaging (SS-EPI) sequence with water excitation and the scanning parameters including repetition time of 4,000 ms, echo time (TE) of 78.1 ms, *b*-values of 0 and 1,000 s/mm^2^, 6 NEXs, FOV of 210 × 210 mm, slice thickness of 10 mm, matrix size of 128 × 128, and bandwidth of 250 kHz. The protocol parameters in IDEAL-IQ and DWI are summarized in Table 2.

**Table 2:** The protocol parameters for IDEAL-IQ and DWI sequences

	IDEAL-IQ	DWI
TR	19.6 ms	4,000 ms
TE	6 echoes (1.12, 3.008, 4.896, 6.784, 8.672, and 10.56 ms)	78.1 ms
NEX	4	4 (*b* = 0), 6 (*b* = 1,000 s/mm^2^)
FOV	220 × 220 mm	210 × 210 mm
Matrix size	128 × 128	128 × 128
Slice Thickness	10 mm	10 mm
FA	5°	90^o^
Bandwidth	90.91 kHz	250.00 kHz
Acquisition time	1 min 38 s	1 min 32 s

*FA* Flip angle, *FOV* Field of view, *NEX* Number of excitations, *TE* Echo time, *TR* Repetition time. See the text for the denomination of the sequences

### Imaging processing

Proton-density fat fraction maps were automatically generated by the MRI scanner. ADC maps were generated via pixel-by-pixel computation from b_0_ and b_1000_ images basing on the Stejskal–Tanner relationship: ADC = ln(*S*_*b* = 0_/*S*_*b* = 1000_)/b. In both DWI and IDEAL-IQ scan, an axial slice, which was perpendicular to the long axis of cylindrical cones and contained the largest cross-sectional area of the phantom, was chosen for region-of-interest selection. Circular region-of-interests with one in each cylindrical cone were placed avoiding the partial volume effect of the cylindrical wall and extra-cylindrical noise. A total of three repetitions, each comprising 30 combos with five GBDs (0, 0.1, 0.25, 0.5, and 1.0 g/50 mL) and six FCs (0, 10, 20, 30, 40, and 50%) (Table 1), were prepared in order to evaluate test-retest reliability among different measures. Thus, each combo of the phantom was measured three times independently.

### Statistical analysis

All values were presented as mean and standard deviations. Linear regression analysis was used to evaluate the relationship among the FF, GBD, and ADC values. Intraclass correlation coefficient and coefficient of variation were used to evaluate test-retest reliability among measures of 3 different phantoms. A *p* value less than 0.05 was considered statistically significant.

## Results

The coefficient of variation was 4.1 and 1.8% for in measuring ADC and FF, respectively. The intraclass correlation coefficient was 0.997 and 0.999 for in measuring ADC and FF, respectively, for three different phantoms, suggestive of excellent reliability as suggested by Koo et al. [28].

**Fig. 2:**
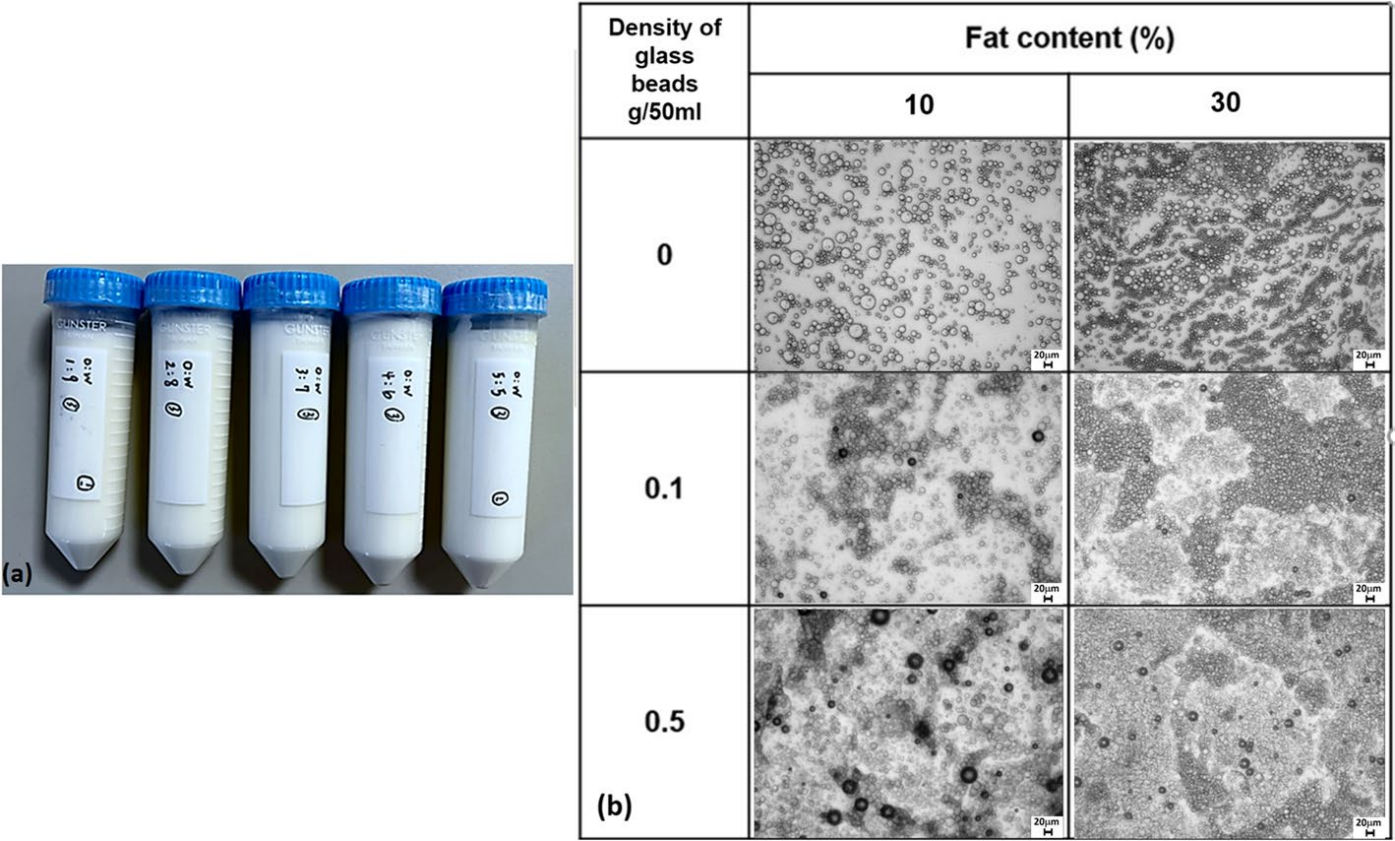
**a** The fat-water phantom housing five cylinders containing agarose-based emulsions with a fat content of 10, 20, 30, 40, and 50% (left to right), respectively, was constructed. Vials are lying horizontally to demonstrate the solid nature of the phantom at room temperature. **b** Micrographs (×40) of 10% of fat content and 30% of fat content with 0, 0.1, and 0.5 g/50 mL of glass bead density, respectively

The gross and microscopic appearances of the proposed phantom are partly demonstrated in Fig. 2. Figure 2a demonstrates 5 cylindrical cones, which contained agarose-based fat-water emulsions with FC of 10, 20, 30, 40, and 50%, respectively. These cylindrical cones were intentionally placed horizontally in order to demonstrate the solidity of these fat-water emulsions at the room temperature. Figure 2b demonstrates six microscopic images mixing GBDs (0, 0.1g/50 mL, and 0.5g/50 mL) and fat-water emulsions (FC 10 and 30%), showing the geometric distribution and arrangement of glass beads, fat droplets, and water in the emulsion.

**Fig. 3:**
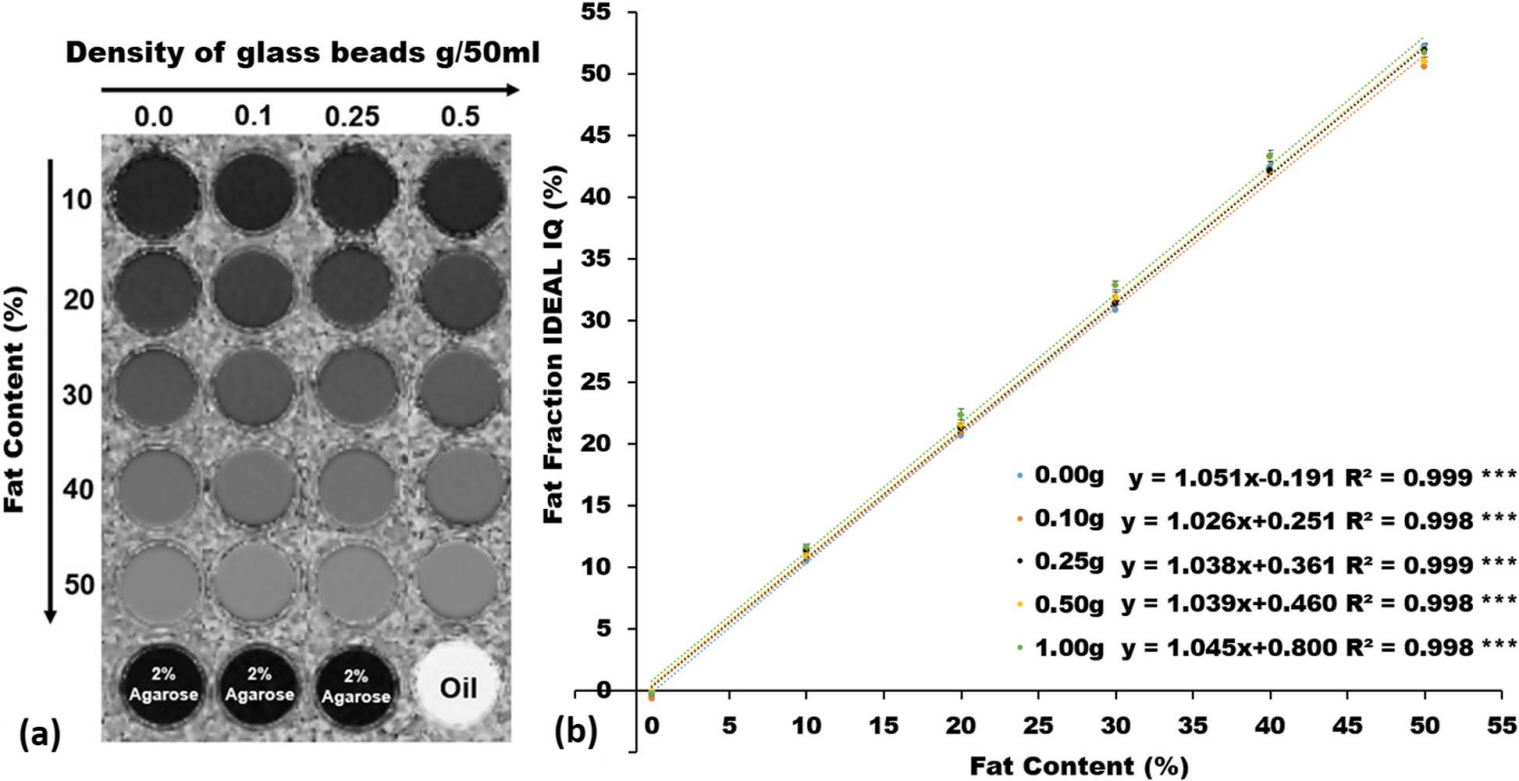
**a** Fat fraction (FF) maps of IDEAL-IQ in 10, 20, 30, 40, and 50% of fat content (top to down) with respect to 0, 0.1, 0.25, and 0.5 g/50 mL of GBD (left to right). **b** Linear relationship between the FF measured by IDEAL-IQ and the fat content of phantom in different GBD of 0, 0.1, 0.25, 0.5, and 1.0 g/50 mL, respectively. *** *p* < 0.001

**Fig. 4:**
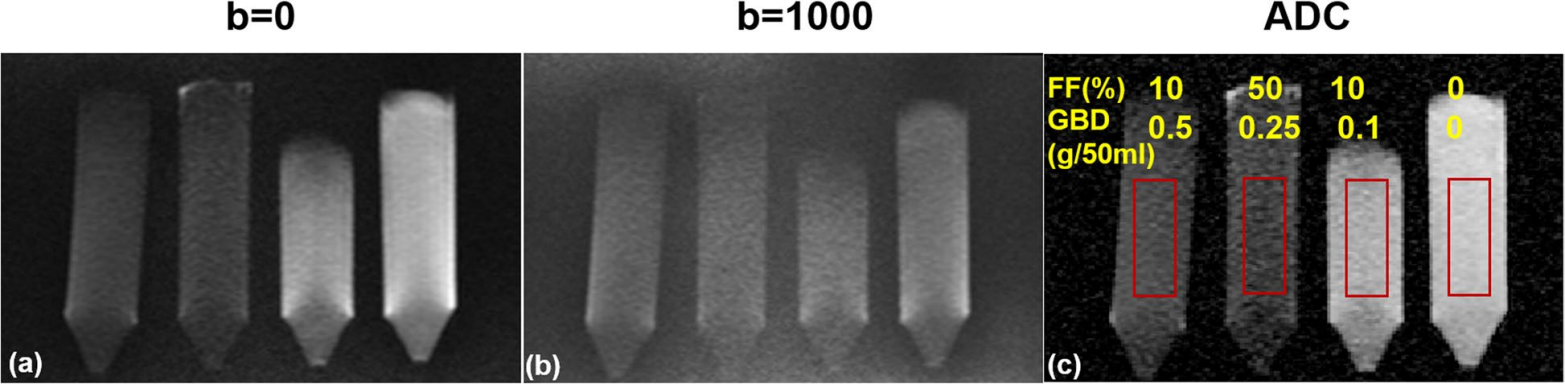
One slice of the diffusion-weighted images: *b* = 0 s/mm^2^ (**a**), *b* = 1,000 s/mm^2^ (**b**), and the ADC map (**c**). The red rectangle indicates the location of the regions of interest. *ADC* Apparent diffusion coefficient

**Fig. 5:**
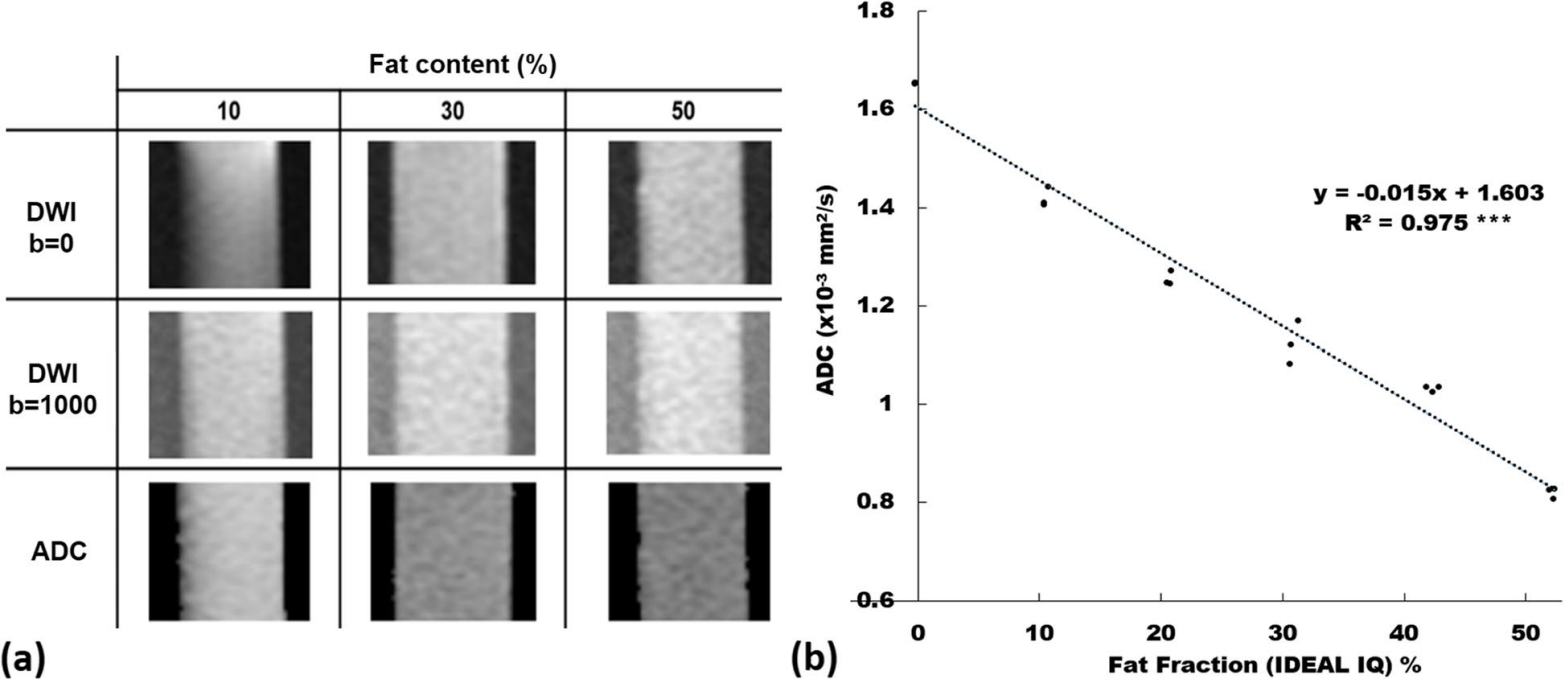
**a** Diffusion-weighted images (*b* = 0 s/mm^2^ and *b* = 1,000 s/mm^2^) and ADC maps of emulsions comprising fat content of 10, 30, and 50% plus 2% agarose and water without adding any glass beads. **b** Scatter plot and linear regression (dotted line) of ADC *versus* fat fraction measured by IDEAL-IQ (see the text for this sequence) among three independently prepared phantoms. *ADC* Apparent diffusion coefficient. *** *p* < 0.001

The imaging appearance and performance in estimating the FC using the IDEAL-IQ under different GBDs were demonstrated in Fig. 3. Figure 3a illustrates the PDFF maps of emulsions with the FF varying from 10 to 50% and the GBD ranging from 0 to 0.5 g/50 mL. Figure 3b demonstrates scatter plots of the FF measured by the IDEAL-IQ and the FC in the originally prepared phantom, showing significantly linear relationship between the FF and the FC (*y* = *ax* + *b* with *slope a* ranging from 1.026 to 1.051, bias *b* ranging from -0.191 to 0.8, R^2^ ranging from 0.998 to 0.999, and all *p* < 0.001) in all GBDs. One slice of DWI and ADC using SS-EP-DWI sequence is shown in Fig. 4. The imaging appearance and performance of the phantom in measuring the ADC value using SS-EP-DWI under different FFs are shown in Fig. 5. Figure 5a shows the cropped DWI (*b* = 0 and 1000 s/mm^2^, respectively) and ADC maps under the FF of 10, 30, and 50%. Figure 5b displays a significant and inverse linear relationship (*y* = −0.015*x* + 1.603, R^2^ = 0.975, *p* < 0.001) between the ADC and the FF without adding any glass beads (GBD = 0).

**Fig. 6:**
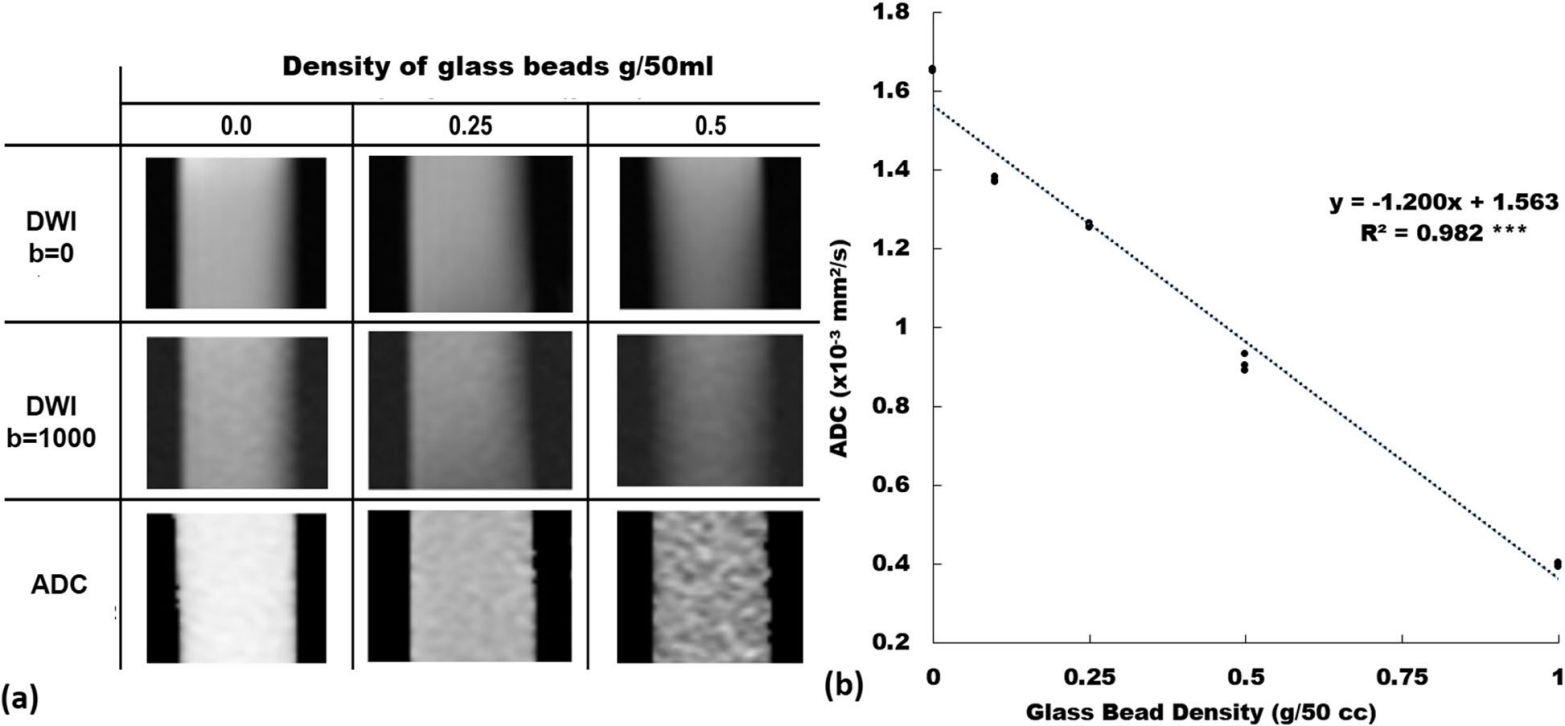
**a** Diffusion-weighted images (*b* = 0 s/mm^2^ and *b* = 1,000 s/mm^2^) and ADC maps with a GBD of 0, 0.25, and 0.5 g/mL in water only (0% of fat content, no agarose). **b** Scatter plot and linear regression (dotted line) of ADC *versus* glass bead density among three independently prepared phantoms. *ADC* Apparent diffusion coefficient, *GBD* Glass bead density. *** *p* <0.001

**Fig 7:**
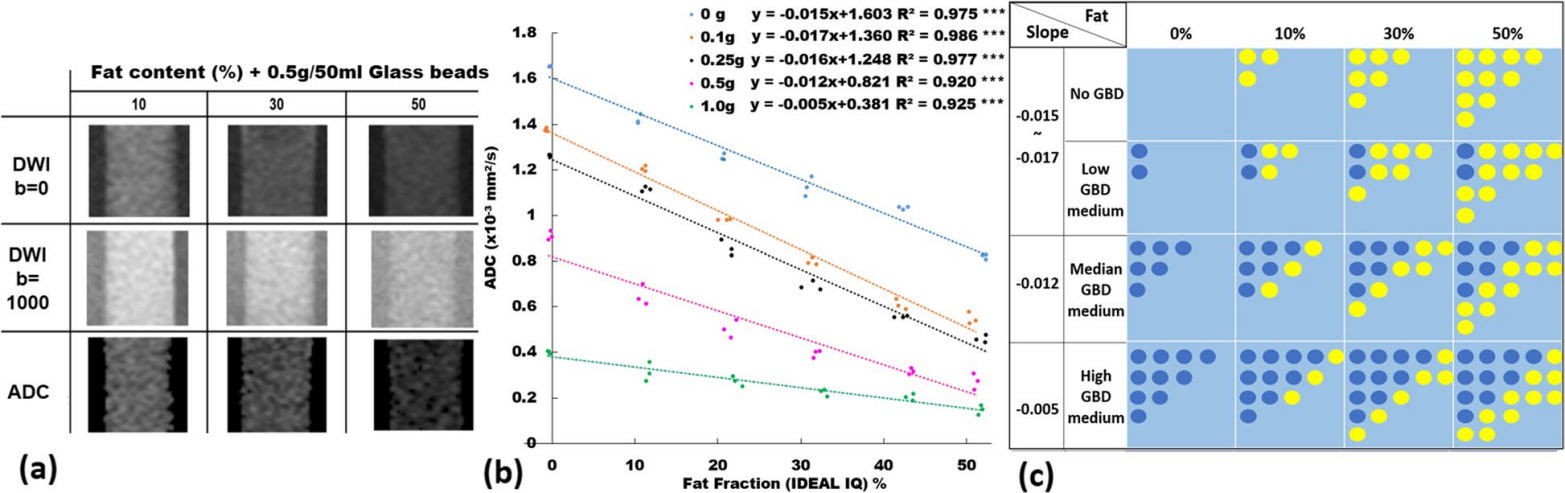
**a** Diffusion-weighted images (*b* = 0 s/mm^2^ and *b* = 1,000 s/mm^2^) and ADC maps with a GBD 0.5 g/mL with fat content of 10, 30, and 50%. **b** ADC values at different GBD (0, 0.1, 0.25, 0.5, and 1.0 g/50 mL) with respect to different phantom fat fractions measured by IDEAL-IQ (at fat content of 0, 10, 20, 30, 40, and 50%). **c** Each box represents a pixel of the image, containing fat droplets (yellow circles), glass beads (dark blue circles), and water outside the fat droplets and glass beads. *ADC* Apparent diffusion coefficient, *GBD* Glass bead density. *** *p* <0.001

The imaging appearance and performance of the phantom in measuring the ADC value using SS-EP-DWI under different GBDs are shown in Fig. 6. Figure 6a shows the cropped DWIs (*b* = 0 and 1,000 s/mm^2^, respectively) and the ADC maps with the GBD of 0, 0.25, and 0.5 g/50 mL, respectively. Figure 6b displays a significant and inverse linear relationship (*y* = -0.12*x* + 1.563, R^2^ = 0.982, *p* < 0.001) between ADC and GBD without FC (= 0). The imaging appearance and performance of the phantom in measuring the ADC value using SS-EP-DWI under different FFs and GBDs are shown in Fig. 7. Figure 7a shows the cropped DWIs (*b* = 0 and 1,000 s/mm^2^) and the ADC maps of the emulsions with FF of 10, 30, and 50%, at the GBD of 0.5 g/50 mL. Figure 7b displays the ADC *versus* FF scatter plots of the emulsions under the GBD of 0, 0.1 g/50 mL, 0.25 g/50 mL, 0.5 g/50 mL, and 1.0 g/50 mL, showing a significant and inverse linear relationship between the ADC and the FF at each GBD (*y* = *ax* + *b*, with *a* ranging from -0.005 to -0.017 and *b* ranging from 0.381 to 1.603, R^2^ = 0.925 to 0.986, all *p* < 0.001), with a decreasing interception as the GBD increases. Figure 7c illustrates the overlapping phenomenon of ADC among emulsions across different GBDs and FFs. For example, emulsions with 0.1 g/50 mL GBDs plus 40−50% FFs shared a same lower ADC range with those with 0.5 g/50 mL GBDs plus 10~20% FFs. Likewise, emulsions with zero GBDs but 50% FFs had a median ADC range overlapped by those with 0.1 g/50 mL GBDs plus 30% FFs and those with 0.25 g/50 mL GBDs plus 20% FFs.

Table 3 shows all ADC values (mean ± standard deviation) of all 30 emulsions within five GBDs (0, 0.1, 0.25, 0.5, and 1.0 g/50 mL) and six FFs (0, 10, 20, 30, 40, and 50%).

**Table 3:** ADC of the proposed fat-containing diffusion phantom

GBD g/50 mL	Fat content (%)					
	0	10	20	30	40	50
0	1.653 ± 0.002	1.419 ± 0.020	1.253 ± 0.014	1.124 ± 0.043	1.031 ± 0.006	0.819 ± 0.011
0.1	1.375 ± 0.007	1.205 ± 0.012	0.980 ± 0.002	0.797 ± 0.016	0.608 ± 0.024	0.547 ± 0.027
0.25	1.261 ± 0.005	1.114 ± 0.010	0.855 ± 0.034	0.691 ± 0.020	0.555 ± 0.003	0.458 ± 0.017
0.5	0.909 ± 0.021	0.648 ± 0.045	0.502 ± 0.038	0.393 ± 0.016	0.315 ± 0.014	0.271 ± 0.035
1.0	0.399 ± 0.006	0.312 ± 0.041	0.272 ± 0.023	0.222 ± 0.016	0.202 ± 0.015	0.145 ± 0.021

*ADC* Apparent diffusion coefficient (×10^−3^ mm^2^/s), *FC* Fat content (%), *GBD* Glass bead density (g/50 mL). The ADC value is presented as mean ± standard deviation

## Discussion

Currently, fat-water phantoms and diffusion phantoms have been used to validate MRI-based methods in quantifying the FC [12, 29–33] and to measure the diffusivity of polyethylene particle suspensions or microbead impregnated gels on DWI [11, 19, 34, 35], respectively. However, these phantoms do not enable us to clarify the impact of FC on the measure of the diffusivity of polyethylene or microbead preparations in any single phantom. Under this circumstance, it remains controversial regarding the role of fat on measuring the diffusivity of a phantom. In humans, the FC measured by the IDEAL method [36] varies across age, gender, and body mass index in parotid glands [24] and spine [37–39], and has a discrepant impact on the measures of ADC, *i.e.*, a negative association between FC and ADC in some studies [24, 37] but a positive association between FC and ADC in other study [39]. To solve the discrepant clinical observations, a dual-function phantom allowing measures of FC and ADC is demanded. To the best of knowledge, however, a dual-function phantom designed to quantify the ADC in different FCs and different cellular densities to simulate the real situation of human tissues has not been documented yet.

In this study, we designed the first dual-function phantom, which not only allows us to quantitatively measure the FC and diffusivity of emulsions, respectively, but also enables us to evaluate the relationship among the GBD, FF, and ADC. By adding the agarose into the emulsion, the emulsion becomes a rigid gel at room temperature, thus successfully prolonging the storage period of the phantom on the one hand and preventing oil-water emulsion from dissociation and glass particles from sinking or suspending on the other hand.

The proposed phantom has two different utilities. First, it simulates the fatty environment of human tissues by preparing agarose-based emulsions with six different FCs of 0, 10, 20, 30, 40, and 50%. Second, it imitates water diffusion of human tissues by preparing five different GBDs (0, 0.1, 0.25, 0.5, and 1.0 g/50 mL). Our results showed that the FF is proportional (slope, 1.02~1.05; R^2^, 0.99) to the FC which is similar to that in Bernard’s study [12]. It is worthy to mention that the IDEAL-IQ provides high accuracy of FF in our phantom irrelevant to the GBD (Fig. 3), meaning that we can infuse different GBDs into the fat-water emulsion without altering the FF. In measuring the diffusivity, our results showed that the ADC decreases as the GBD increases similar to that reported in Portakal’s study [11]. The negative association between the ADC and the FF and between the ADC and the GBD can be attributed to the space for diffusion, which reduces as the FF and the GBD increases.

DWI is a technique that quantifies the water diffusion, reflects the degree of restricted water mobility in biological tissues, and characterizes the relation of the diffusivity with cellularity. Therefore, ADC has been widely used as a biomarker for early diagnosis [40], evaluation of tumor differentiation [41], tumor grade [42], and treatment response [43] of malignancy. Some researchers have reported that the ADC measurement is influenced by the adipose tissue in breast [23], bone [39], and parotid gland [24], attributing to the characteristics of limited diffusion in fatty tissue. However, the potential mixed effect of cellularity and FC on the ADC measurement has not been investigated so far. Our results demonstrated that the effect of the FC and the GBD on the ADC is additive. With a GBD of 0, the ADC is negatively and linearly associated with the FF. Likewise, the ADC is negatively and linearly associated with the GBD with a FF of 0. When glass beads are added, the slope of the linear relationship between the ADC and the FF, however, varies among different GBDs, *i.e.*, the higher the GBD, the lower the slope, suggesting an additional effect of the GBD. The negative association between ADC and FF in our results is consistent with the findings in Hansmann et al.’s study [44]. Hansmann’s study indicated that reduced ADCs by FF varied at different *b*-values, and our results further reveal that the slope of reduced ADCs with increased FF at *b* = 1,000 is influenced by the initial ADCs with GBD.

It is well known that echo-planar sequences are equipped by fat-suppressed techniques in order to prevent chemical shift ghost artifacts and potential underestimation of ADC [45]. By using fast spin echo “PROPELLER” DWI with fat saturation and without fat saturation, Juan et al. also disclosed that the underestimation of ADC by the non-fat-saturated DWI sequence could be remedied by fat-saturated DWI sequence [46]. Therefore, the influence of fat on the ADC measurement on SS-EP-DWI is usually neglected. Reduced extravascular and extracellular space has been considered to be one of the causes of alternation of tissue perfusion [47]. Results of microscopic examination of our phantom study clearly demonstrate the gradual reduction of space outside the fat droplets and glass beads parallel to the increased amount of fat droplets and glass beads step by step. This observation supports the assumption that the reduced space of diffusion as the main cause of the inverse relationship between ADC and FC and between ADC and GBD. Our results showed reduced diffusion.

Since that both fat droplets and glass beads occupy the space for diffusion of water, it deserves our attention to better characterize the impact of the FF and the GBD on the ADC measurement. First, the ADC is not determined by either the FF or the GBD alone but influenced by the FF and the GBD together. Second, the ADC does not represent the GBD specifically. A GBD with different FFs might have different ADCs. On the other hand, an ADC might be found in different GBDs due to the influence of different FFs. Third, the ADC reflects the GBD with a negative linear association whenever the FF is fixed. Accordingly, to avoid the fat-related bias in the comparison of ADC among different studies and the use of ADC as a biomarker, it is essential to obtain the FF first.

Understanding the relationship among the FF, GBD, and ADC is clinically relevant especially in the fat-rich organs such as breast, liver, and spine as well as in the fat-containing tumors such as lipoma, lipoblastoma, angiolipoma, spindle cell lipoma, pleomorphic lipoma, myolipoma, chondroid lipoma, lipomatosis, hibernoma, fat necrosis, well-differentiated liposarcomas and atypical lipomatous tumors, hepatocellular carcinoma, regenerative nodule, hepatocellular adenoma, focal nodular hyperplasia, and steatosis [48, 49]. By taking the FF into account, clinicians are able to interpret the ADC of a lesion and avoid false positive interpretation as previously encountered [25].

There are some limitations in our study. First, we conducted this study only using the SS-EP-DWI, which has been widely used in daily clinical practice, at a field strength of 1.5 T with a diffusion gradient of *b* =1,000 s/mm^2^. Hansmann’s study [44] evaluated the variation in ADC at different *b*-values using fat-water phantoms (FF 0%, 20%, 30%, 50%). They found that choice of *b*-values impacts predicted ADC when fat is present in tissues, and these differences increase with higher FF. In this study, we only employed the mono-exponential DWI model with *b*-values of 0 and 1,000 s/mm^2^. Factors related to the pulse sequence, *b-*value, acceleration factor, fat saturated or not, and field strength, which have been shown to influence the measures of ADC [46, 50], were not investigated in the current study. Further studies designed with different pulse sequences, *b*-value, acceleration factors, fat-saturated conditions, and field strengths to disclose the relationship among the FF, GBD, and ADC are warranted. Nevertheless, we have successfully shown the complex relationship among the FF, GBD, and ADC in this study. Second, the diffusion model was based on the assumption of Gaussian distribution with the ADC calculated based on a mono-exponential equation rather than non-mono-exponential diffusion models, such as kurtosis, stretched exponential, and statistical models. It deserves further studies to evaluate the potential heterogeneity of the proposed phantom. Third, the GBD used in this study is made of hollow glass microspheres with diameters ranging from 20 to 80μm, different from to human cells by the nature of compositions and size. In order to better simulate the human tissue, we have launched another study using a phantom composed water-filled, lipid bilayers to simulate the human cells to examine the impact of the FC on the ADC measures at different densities of lipid bilayers.

In conclusion, a novel dual-function phantom equipped by different FCs and GBDs was proposed to simulate the human tissue environment and to allow us better quantifying the influence of the FC and GBD on the ADC measures in future clinical practice.

## Data Availability

The authors confirm that the data and materials supporting the findings of this study are available on request.

## Abbreviations

ADC: Apparent diffusion coefficient
DWI: Diffusion-weighted imaging
FC: Fat content
FF: Fat fraction
GBD: Glass bead density
IDEAL-IQ: Iterative decomposition of water and fat with echo asymmetry and least squares estimation-IQ
MRI: Magnetic resonance imaging
RPM: Revolution per minute
SS-EP-DWI: Single-shot echo-planar diffusion-weighted imaging
SS-EPI: Single-shot echo-planar imaging
